# Dipeptidyl peptidase 4 inhibitor sitagliptin decreases myocardial fibrosis and modulates myocardial insulin signaling in a swine model of chronic myocardial ischemia

**DOI:** 10.1371/journal.pone.0307922

**Published:** 2024-07-29

**Authors:** Dwight D. Harris, Sharif A. Sabe, Mark Broadwin, Chris Stone, Krishna Bellam, Akshay Malhotra, M. Ruhul Abid, Frank W. Sellke

**Affiliations:** Division of Cardiothoracic Surgery, Department of Surgery, Cardiovascular Research Center, Rhode Island Hospital, Alpert Medical School of Brown University, Providence, RI, United States of America; Indiana University School of Medicine, UNITED STATES OF AMERICA

## Abstract

Although both clinical data and animal models suggest cardiovascular benefits following administration of Dipeptidyl Peptidase 4 (DPP-4) inhibitors, the underlying mechanisms remain unclear. We therefore sought to evaluate the effect of the DPP-4 inhibitor sitagliptin on myocardial fibrosis, and insulin signaling in chronic myocardial ischemia using a swine model. An ameroid constrictor placement on the left coronary circumflex artery of thirteen Yorkshire swine to model chronic myocardial ischemia. After two weeks of recovery, swine were assigned to one of two groups: control (CON, n = 8), or sitagliptin 100mg daily (SIT, n = 5). After 5 weeks of treatment, the swine underwent terminal harvest with collection of myocardial tissue. Fibrosis was quantified using Masson’s trichrome. Protein expression was quantified by Immunoblotting. Trichrome stain demonstrated a significant decrease in perivascular and interstitial fibrosis in the SIT group relative to CON (all p<0.05). Immunoblot showed a reduction in Jak2, the pSTAT3 to STAT 3 Ratio, pSMAD 2/3, and SMAD 2/3, and an increase in STAT 3 in the SIT group relative to CON (all p<0.05). SIT treatment was associated with increased expression of insulin receptor one and decreased expression of makers for insulin resistance, including phospho-PKC- alpha, RBP-4, SIRT1, and PI3K (p<0.05). Sitagliptin results in a reduction in perivascular and interstitial fibrosis and increased insulin sensitivity in chronically ischemic swine myocardium. This likely contributes to the improved cardiovascular outcomes seen with DPP-4 inhibitors.

## Introduction

Despite the status of chronic coronary artery disease (CAD) as a leading cause of mortality and morbidity worldwide, therapeutic options remain limited for patients deemed unsuitable for surgical or endovascular revascularization [1–3]. Given this, there is growing interest in the development of novel therapies for the management of advanced CAD. Among these, dipeptidyl peptidase 4 (DPP-4) inhibitors have been the subject of particularly extensive investigation. DPP-4 inhibitors function by inhibiting DPP-4, a proteolytic enzyme that inactivates glucose‐lowering incretins [4].

DPP-4 inhibitor administration has yielded mixed results in treating patients with CAD and heart failure in both pre-clinical and clinical studies, with many clinical studies showing no significant change in cardiovascular mortality or related complications [5–11]. While the SAVOR-TIMI-53 clinical trial showed increased heart failure hospitalizations, this finding has not been redemonstrated in other clinical studies, and a recent study from Japan showed better long-term outcomes in heart failure patients treated with DPP-4 inhibitors [11–13]. Pre-clinically, multiple small and large animal models have shown improved cardiac outcomes with DPP-4 inhibitor treatment. In small animal models, DPP-4 inhibitors improve left ventricular functional recovery, reduce infarct size, reduce apoptosis, and attenuate endothelial proliferation [14–19]. We have previously shown, using our validated swine model for chronic CAD, that the DPP inhibitor sitagliptin (SIT) increased in myocardial perfusion and arteriolar collateralization in the ischemic myocardium [20]. There was no change in cardiac output, makers for systolic function, and markers for diastolic function [20]. This study was complicated by a 50% mortality in the SIT group compared to a historical 20% mortality in the control (CON) group [20]. This is possibly related to a decrease in cardiac index and decreased oxidative phosphorylation in the SIT group; however, more work is needed to validate this finding [20, 21].

While the evidence to support the use of DPP-4 inhibitors continues, as above, to grow, the underlying molecular mechanisms are unclear. It has been well demonstrated that DPP-4 inhibitors reduce fibrosis and modulate insulin signaling in several pathways; however, our study represents the first attempt to study the changes in myocardial fibrosis and insulin signaling with SIT in a swine model of chronic myocardial ischemia.

## Methods

### Swine model

This study is an additional analysis of our previously described cohort [20]. Thirteen Yorkshire swine (Tufts University Farm, North Grafton, MA, USA) underwent ameroid constrictor (Research Instruments SW, Escondido, CA, USA) placement on the left coronary circumflex artery (LCx) to induce chronic myocardial ischemia. After two weeks of recovery, animals received either CON (n = 8, F = 3, M = 5), or Sitagliptin 100mg daily (SIT, n = 5, F = 1, M = 4)) (Merck & Co, Rahway, New Jersey, USA). After 5 weeks of treatment, swine underwent terminal harvest with collection of myocardial tissue from the ischemic and non-ischemic area.

### Humane animal care

Animals were cared for in compliance with current ethical standard including the Principles of Laboratory Animal Care and the Guide for the Care and Use of Laboratory Animals. The Rhode Island Hospital Institutional Animal Care and Use Committee approved and monitored this protocol (Protocol #505821)(20). The ameroid constrictor model is known to have a mortality up to 20%. This mortality is normally at the 2 week time point due to ameroid closure causing sudden cardiac death. We do not consider mortality to be a primary endpoint of the study as historically our mortality rate is lower than expected at 15–17% and historical most deaths are before starting drug therapy. Given most deaths in the study are from sudden cardiac death (Over 95% of deaths in the last two years) and our mortality rate is consistently below the expected 20% this study does not have strictly defined humane endpoints. The Rhode Island Hospital Institutional Animal Care and Use Committee has committee specifically reviewed and approved the anticipated mortality in the study design and the use of soft humane endpoints. The animals used in this study are monitored daily by animal care staff and veterinary technicians. The animals are examined a minimum of twice per day by staff in the animal facility. In the rare event that an animal is found to be in distress (shortness of breath, signs of unexpected pain, poor oral intake, or difficulty with ambulation), an immediate evaluation is performed by both the attending veterinarian and the senior lab staff. The animal is examined and indicated work up performed. The goal is always to minimize suffering and distress while avoiding unnecessary mortality. The team discusses possible diagnoses and treatment. The decision to move forward with euthanasia is based on a joint discussion with the veterinarian and the senior lab staff. If it is decided that euthanasia is the only option to minimize suffering and distress it is performed within the hour.

### Protocol interruption

The protocol originally had ten animals allotted to the sitagliptin group, however, the SIT group had, as previously described, a 50% mortality rate [20]. The CON group was composed of four female pigs, of which one died intraoperatively due to ventricular fibrillation [20]. Five male pigs are from a historical cohort, that used an identical surgical technique, due to tissue availability and the goal to minimize animal utilization [20]. This cohort had a 17% mortality rate with all deaths due to sudden cardiac death [20]. The SIT group was composed of 10 pigs (five 5 and 5 male). there were a total of five deaths (four females, one male, 50% mortality) [20]. Three female pigs were found dead in their cage several weeks after ameroid placement and initiation of drug therapy, with unclear etiology on necropsy and presumed sudden cardiac death [20]. One female pig was found to be lethargic, and in respiratory distress and was euthanized. One male pig was found dead on the morning of postoperative day 1 with cardiac tamponade as the suspected cause of death [20]. Due to the high mortality rate in the treatment group, the Animal Care and Use Committee temporarily paused treatment in two of the animals for two weeks given the mortality [20]. After review, the study was allowed to continue by giving the remaining two swine a one-week course of SIT prior to harvest [20].

### Ameroid constrictor placement

Preoperative care and anesthesia were performed as previously described [22]. The swine were placed in right lateral decubitus position and prepped and draped in a sterile fashion. A left thoracotomy was performed at the second intercostal space as previously described. The left coronary circumflex (LCx) was exposed at its takeoff from the the left main coronary artery. The LCx was circled with a vessel loop. The area at risk for ischemia was mapped by occluding the LCx for two minutes while injecting a 5 ml solution of gold microspheres (BioPal, Worcester, MA). Ischemia was confirmed by t-wave changes on the EKG. The ameroid constrictor was placed at the base of the LCx to create consistent infarcts. The surgical site was closed in layers with absorbable suture as previously described [22].

### Harvest and functional measurements

Preoperative care and anesthesia were performed as previously described [22]. A median sternotomy was performed and the heart freed from pericardial and sternal adhesions. The swine were euthanized and the heart was sectioned into ischemic and non-ischemic regions and snap frozen in liquid nitrogen. Gold microsphere analysis was used to confirm the most ischemic and least ischemic tissue for analysis.

### Immunoblotting

Lysates were made from the from ischemic and non-ischemic myocardial tissue as previously described(23). Protein quantification performed using Immunoblotting as previously described [23]. NIH Image J software was used for Immunoblot analysis.

### Antibodies

Primary antibodies to GAPDH (Glyceraldehyde 3-phosphate dehydrogenase), Janus kinase 2 (JAK 2), phospho-protein kinase C alpha (pPKCα), phospho-mammalian target of rapamycin (pmTOR), vimentin, mammalian target of rapamycin (pmTOR), phosphoinositide 3-kinases (PI3K), phospho-forkhead box O1 (pFOX01), forkhead box O1 (FOX01), phospho-insulin receptor substrate-1 (pIRS-1), insulin receptor substrate-1 (IRS-1), retinol binding protein 4 (RBP4), and NAD-dependent deacetylase sirtuin-1 (SIRT1), metalloproteinase 13 (MMP13), Filamin A, connexin-43, tissue inhibitor of metalloproteinase 2 (TIMP2), transforming growth factor beta (TGF-Beta), transforming growth factor beta receptor (TGF-BR), mothers against decapentaplegic homolog 2/3 (SMAD 2/3), signal transducer and activator of transcription 3 (STAT3), and phosphorylated STAT3 (p-STAT3) were obtained from Cell Signaling (Danvers, Massachusetts, USA). Primary antibody to phospho-mothers against decapentaplegic homolog 2/3 (pSMAD 2/3) was obtained from Abcam (Cambridge, England, UK).

### Histochemistry

Masson’s trichrome and Terminal deoxynucleotidyl transferase dUTP nick end labeling (TUNEL) stain was performed on ischemic myocardial tissue and imaged by iHisto (iHisto, Salem, MA, USA). Slides were analyzed using QuPath (University of Edinburgh, Edinburgh, Scotland, UK) as previously described [22–24].

### Statistical analysis

All statistical analysis was performed using Prism 9 (GraphPad Software, San Diego, CA, USA). Normality was determined using Shapiro-Wilk test. Data that followed a normal distribution was analyzed using Student’s t-test or one-way analysis of variance (ANOVA), and non-parametric data was analyzed with the Mann–Whitney U test or the Kruskal–Wallis one-way ANOVA. Multiple comparisons in the one-way ANOVA were corrected for using the Bonferroni method, and multiple comparisons in the Kruskal–Wallis one-way ANOVA were corrected for using Dunn’s method. Data is presented as mean and standard deviation. Immunoblot data is shown as mean fold change of the band intensity normalized to the average control for fibrosis and average non-ischemic control for insulin signaling. We excluded outliers more than two standard deviations from the mean.

## Results

### Fibrosis and apoptosis

**Fig 1:**
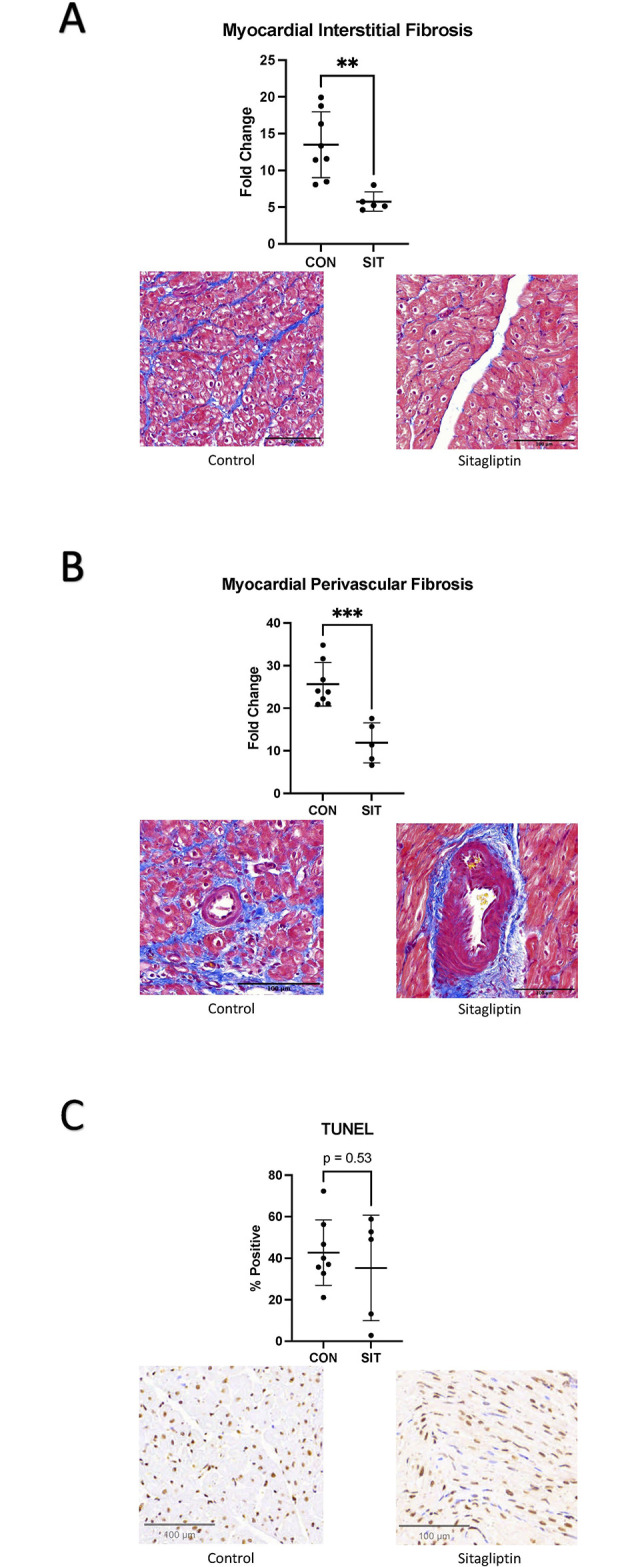
Fibrosis and TUNEL staining. **A:** Myocardial interstitial fibrosis is decreased in the sitagliptin (SIT) compared to the control (CON) group. **B:** Myocardial perivascular fibrosis is decreased in the sitagliptin group compared to control. **C: There was no difference in TUNEL stanning between the groups.** **p<0.01,***p<0.001.

Trichrome staining demonstrated a decrease in perivascular and interstitial fibrosis in the ischemic SIT myocardium (SIT-I) compared to the ischemic CON myocardium (CON-I) (Fig 1, p<0.001, p = 0.003). There was no change in apoptosis by tunnel stanning between groups.

**Fig 2:**
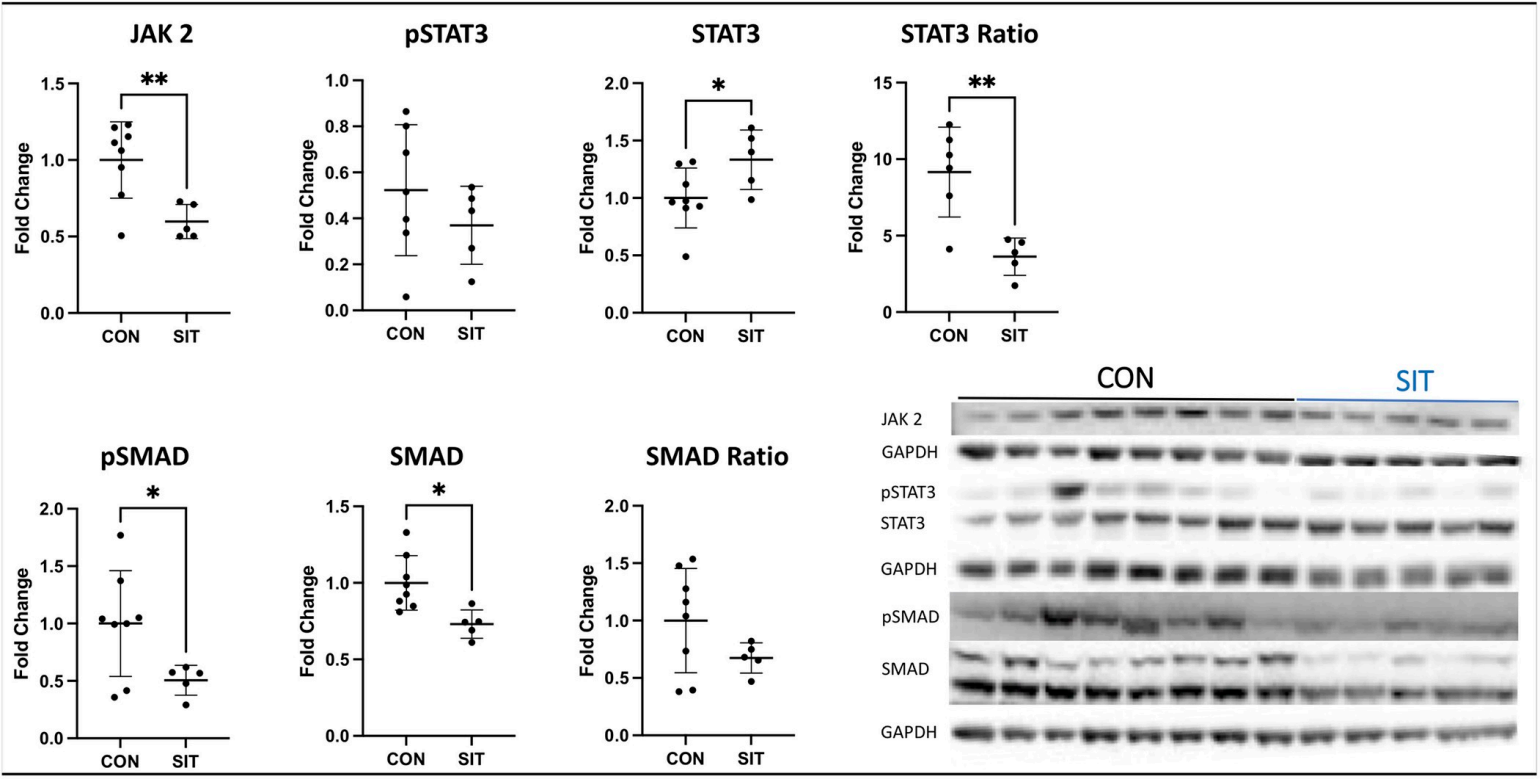
Immunoblotting for JAK, STAT, and SMAD. Janus kinase 2 (JAK 2) was significantly decreased in the sitagliptin (SIT) compared to the control (CON) group. There was a significant decrease in STAT3 and the ratio of pSTAT3 to STAT3 in the SIT group compared to CON. There was also a significant decrease in SMAD and pSMAD in the SIT group. Data points are mean fold change after normalization to average control. *<0.05, **p<0.01,***p<0.001.

**Fig 3:**
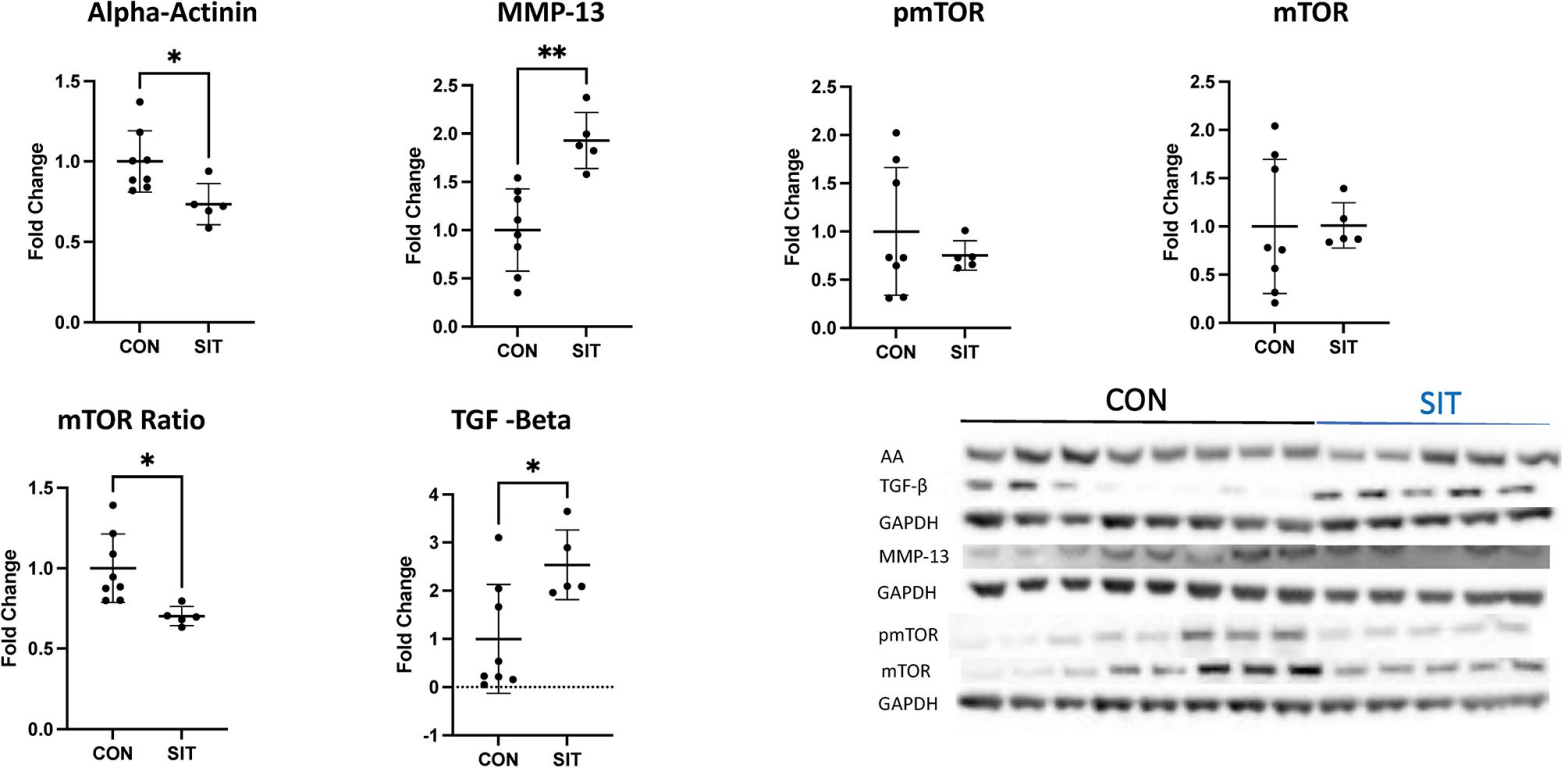
Immunoblotting for TGF-beta, alpha-actinin, MMP-13, and mTOR. Alpha-Actinin (AA) was significantly decreased in the sitagliptin (SIT) compared to the control (CON) group. There was a significant increase MMP-13 and TGF-Beta in the SIT group. There was a significant decrease in the ratio of pmTOR to mTOR. Data points are mean fold change after normalization to average control. *<0.05, **p<0.01,***p<0.001.

Immunoblot showed a decrease in Jak2, the pSTAT3 to STAT 3 Ratio, pSMAD 2/3, SMAD 2/3, the pmTOR to mTOR ratio, and alpha-actinin in the SIT-I myocardium compared to CON-I (Fig 2, all p<0.05). There was a significant increase in STAT 3, MMP-13 and TGF-Beta in the SIT-I myocardium compared to CON-I (Fig 3, all p<0.05). There was no change in pSTAT, connexin, vimentin, filamin A, TIMP 2, and TGF-beta receptor (Table 1, all p>0.05)

**Table 1:** Fibrosis markers. Table shows fibrosis markers. Data points are mean fold change after normalization to average control. Data is shown as mean and standard deviation (SD). Significant p-values (p<0.05) are highlighted in bold.

	SIT (Mean ± SD)	p
Jak2	0.60 ± 0.10	**0.006**
pSTAT	0.37 ± 0.15	0.43
STAT	1.33 ± 0.23	**0.046**
pSTAT Ratio	3.62 ± 1.22	**0.004**
Connexin	0.91 ± 0.06	0.45
Alpha-Actinin	0.81 ± 0.13	**0.02**
Vimentin	0.96 ± 0.24	0.83
Filamin A	0.96 ± 0.12	0.17
TGF-Beta	2.54 ± 0.65	**0.03**
TGF-BR	0.74 ±0.26	0.27
pSMAD 2/3	0.51 ± 0.12	**0.04**
SMAD 2/3	0.73 ± 0.08	**0.01**
pSMAD Ratio	0.67 ± 0.13	0.15
pmTOR	0.75 ± 0.14	0.43
mTOR	1.01 ± 0.21	0.98
pMTOR Ratio	0.70 ± 0.06	**0.01**
TIMP 2	0.89 ± 0.32	0.45
MMP-13	1.93 ± 0.26	**0.001**

### Insulin signaling

**Fig 4:**
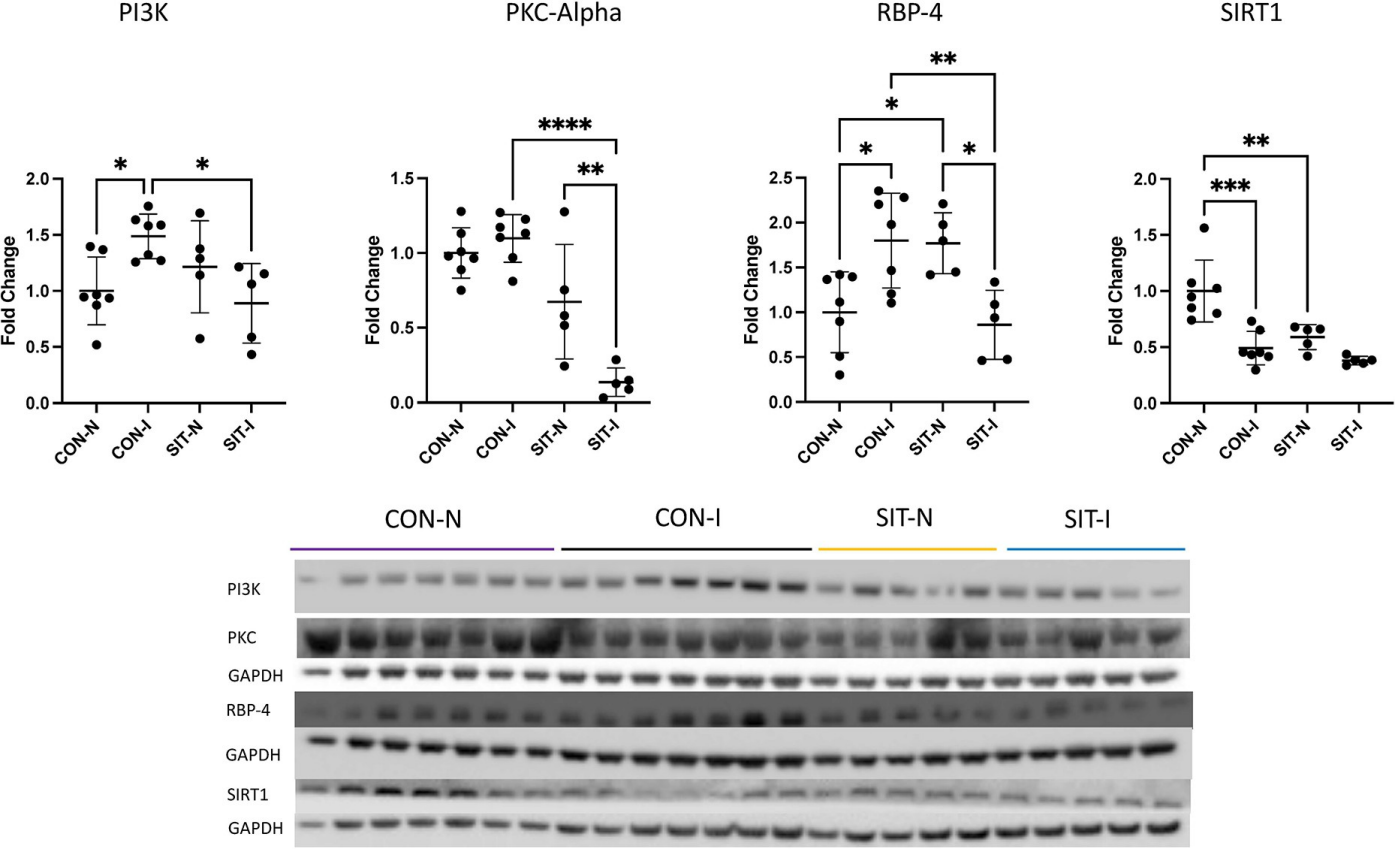
Immunoblotting for insulin sensitivity markers. There was a significant increase in RBP-4 from the control non-ischemic group (CON-N) to the control ischemic group (CON-I). RBP-4 was increased in the sitagliptin non-ischemic group (SIT-N) compared to CON-N. There was a significant decrease in RBP-4 in the sitagliptin ischemic (SIT-I) myocardium compared to CON-I to levels similar to the CON-N myocardium. PI3K was significantly increased in CON-I compared to CON-N. There was a significant decrease in PI3K in SIT-I compared to CON-I similar to CON-N. SIRT1 was significantly decreased in CON-I and SIT-N compared to CON-N. pPKC alpha was significantly decreased in SIT-I compared to CON-I. There was also a significant decrease in pPKC alpha in the SIT-I myocardium compared to SIT-N. Data points are mean fold change after normalization to average control. *<0.05, **p<0.01,***p<0.001.

**Fig 5:**
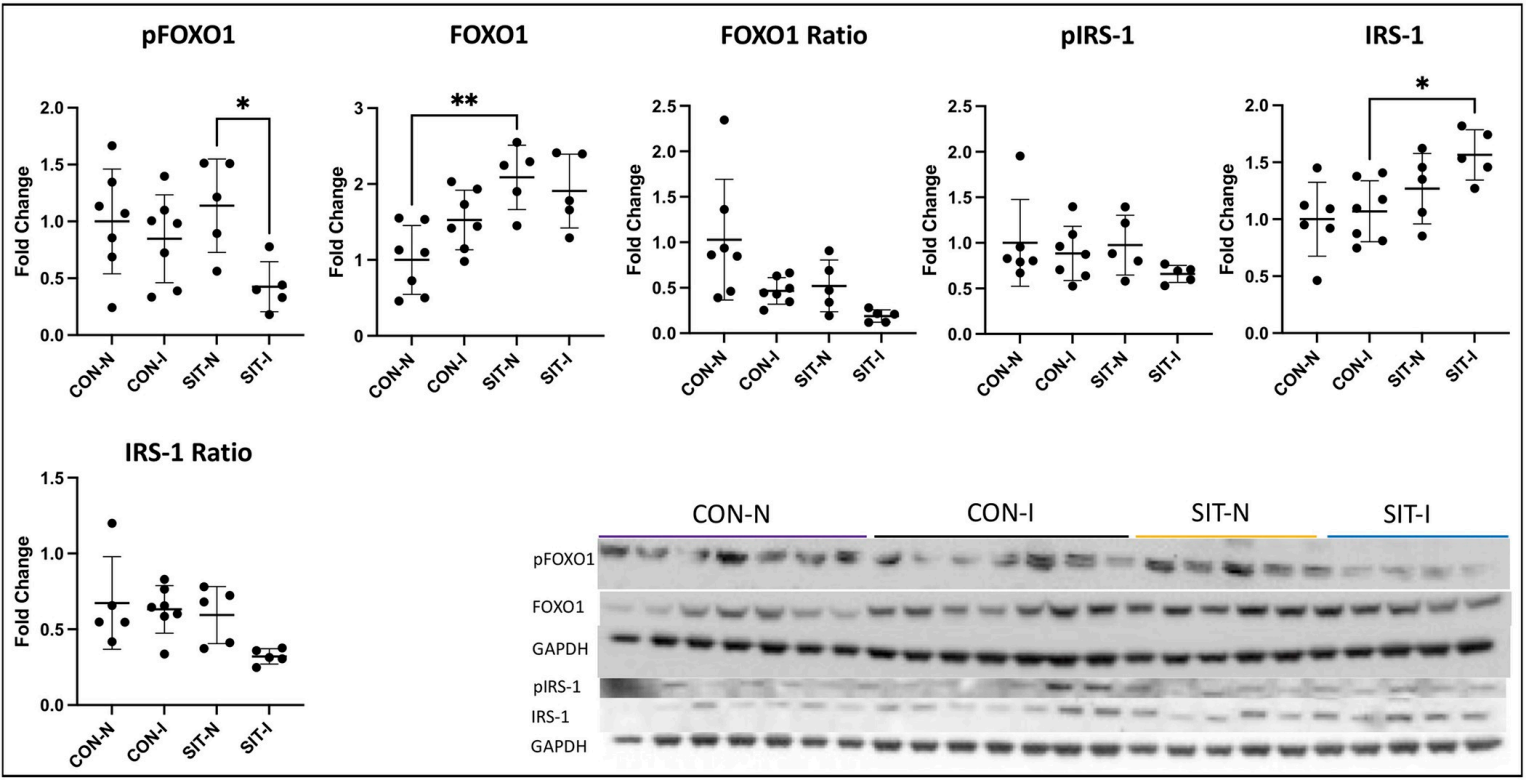
Immunoblotting FOXO1 and insulin receptors. FOXO1 was significantly increased in the sitagliptin non-ischemic group (SIT-N) compared to the control non-ischemic (CON-N) myocardium. pFOXO1 was significantly decreased in the sitagliptin ischemic group (SIT-I) compared to SIT-N. IRS-1 was significantly increased in the SIT-I myocardium compared to the control ischemic group (CON-I). There was no significant change in pIRS-1, pIRS-1 to IRS-1 ratio, or the pFOXO1 to FOXO1 ratio. Data points are mean fold change after normalization to average control. *<0.05, **p<0.01,***p<0.001.

There was a significant increase in RBP-4 from the CON non-ischemic (CON-N) to the CON-I (p = 0.01). RBP-4 was increased in SIT non-ischemic (SIT-N) compared to CON-N (p = 0.03). There was a significant decrease in RBP-4 in the SIT-I compared to CON-I (p = 0.007) to levels similar to CON-N. PI3K was significantly increased in CON-I compared to CON-N (p = 0.04). There was a significant decrease in PI3K in SIT-I compared to CON-I (p = 0.02) similar to CON-N. SIRT1 was significantly decreased in CON-I and SIT-N compared to CON-N (p<0.001, p = 0.003). pPKC alpha was significantly decreased in SIT-I compared to CON-I (p<0.001). There was also a significant decrease in pPKC alpha in the SIT-I myocardium compared to SIT-N (p = 0.004, Fig 4). FOXO1 was significantly increased in SIT-N compared to CON-N (p = 0.002). pFOXO1 was significantly decreased in SIT-I compared to SIT-N (p = 0.04). IRS-1 was significantly increased in the SIT-I myocardium compared to CON-I (p = 0.03, Fig 5). There was no change in pIRS-1, the pIRS-1 to IRS-1 ratio, or the pFOXO1 to FOXO1 ratio (Figs 4, 5).

## Discussion

In our swine model, DPP-4 inhibitor treatment was associated with significant decreases in both interstitial fibrosis and perivascular fibrosis on Masson’s trichrome stain. This reduction in fibrosis was accompanied by a significant decrease in several signaling molecules associated with fibrosis. The JAK/STAT pathway is a widely recognized and extensively studied profibrotic signaling pathway [25]. The SIT group was found to have a significant decrease in JAK 2 and the ratio of pSTAT 3 to STAT 3, in accordance with decreased overall fibrosis.

The TGF-beta and SMAD 2/3 pathway represents another firmly established and extensively researched profibrotic signaling pathway [26]. Our study showed a significant increase in TGF-beta with a paradoxical decrease in pSMAD 2/3 and SMAD 2/3. This could imply that TGF-beta is not activating pSMAD 2/3 at the receptor level, and the increased levels of TGF-beta could be mediating other functions, such as reducing inflammation [27]. Activation of mTOR signaling has been linked to several different types of fibrosis [28–30]. The SIT group had a significant decrease in the ratio of active pmTOR to total mTOR.

MMP-13 is a protease known for its ability to reduce fibrosis in mice lung injury models [31]. Interestingly, the SIT group exhibited an increase in MMP-13, indicating a potential downstream upregulation of antifibrotic proteases. Additionally, the SIT group demonstrated a decrease in alpha-actinin, a cytoskeletal protein that serves as a potential marker for fibrosis [32]. The collective evidence from these signaling molecules and end markers suggests that SIT treatment leads to decreased fibrosis, possibly through the involvement of multiple pathways.

RBP-4, a known marker for insulin resistance, was increased in the CON-I and SIT-N groups compared to CON-N [33]. However, there was a significant decrease in RBP-4 levels in the SIT-I group compared to CON-I, reaching levels similar to CON-N. This indicates that SIT is functioning in both the ischemic and non-ischemic myocardium, with differing effects based on ischemia.

PI3K, a key regulator of insulin signaling, was significantly increased in the CON-I group compared to CON-N [34]. Conversely, PI3K was significantly decreased in the SIT-I group compared to CON-I, reaching levels similar to CON-N. SIRT1, a marker for insulin sensitivity, was significantly decreased in both the CON-I and SIT-N groups compared to CON-N [35]. pPKC alpha, which is known to be upregulated in models of diabetes and insulin resistance, was significantly decreased in the SIT-I group compared to CON-I [36, 37]. IRS-1 was significantly increased in the SIT-I myocardium compared to CON-I.

The results of our study suggest that SIT treatment results in decrease in the markers for insulin resistance and increase in the markers for insulin sensitivity. This likely represents a trend towards normalized insulin signaling by SIT in the ischemic myocardium. The changes in the non-ischemic SIT myocardium, however, are not as straightforward. The SIT group had some changes favoring increased insulin resistance compared to the SIT-I. This may imply that SIT has a global effect on the myocardium that is modulated by ischemia.

It is important to note that with the increased mortality in the SIT group the reduction in fibrosis and STAT could have potentially negative consequences [38–40]. It is well established that fibrosis is a maladaptive stress response that is important in responding to myocardial ischemia [40]. It is possible that loss of this somehow resulted in increased mortality. It should also be noted that cardiac STAT has been shown to have both protective and detrimental effects [38, 39]. STAT has been shown to increase fibrosis, but it has also been shown to decrease oxidative stress and pay roles in ischemic preconditioning [38, 39]. It is possible that loss of STAT resulted in detrimental changes and increased mortality. This is limited to speculation as most animals died suddenly overnight, and the tissue was not reliable for testing.

In summary, our study demonstrates that SIT treatment in a swine model of chronic myocardial ischemia leads to a notable reduction in both interstitial and perivascular fibrosis in the ischemic myocardium. This favorable outcome is accompanied by significant alterations in key fibrotic pathways—namely JAK/STAT, SMAD, and mTOR—in the SIT-treated ischemic myocardium. Furthermore, SIT treatment produced a clear improvement in insulin signaling, as was indicated by decreased markers for insulin resistance and increased markers for insulin sensitivity. This positive response likely signifies a progressive normalization of insulin signaling in the ischemic myocardium. The observed decline in fibrosis and enhancement in insulin signaling may contribute to the previously noted improvement in cardiac function seen in both our earlier study and in human clinical trials; these promising findings shed light on the potential mechanisms through which SIT treatment could exert beneficial effects on cardiac function in the context of chronic myocardial ischemia.

This study represents a substantial advancement in our understanding of the mechanisms underlying DPP-4 inhibitors in the context of chronic myocardial ischemia. Nevertheless, there are several limitations that warrant future considerations. One notable limitation is the small sample size, resulting from significant mortality within the SIT group. This may have compromised the study’s ability to detect more subtle changes between the treatment groups. Additionally, the observed significant mortality in the treatment group could have acted as a selective force, allowing only those swine with the most favorable response potential to survive. This, in turn, may have influenced the overall outcome and interpretation of the results. Another limitation pertains to the interruption in treatment for two of the swine, as this potentially masked long-term changes that could have occurred during the pause in treatment.

Furthermore, it is important to acknowledge that chronic myocardial ischemia often exhibits gender-specific phenotypes. However, due to the mortality within the SIT group, our study lacked the power to thoroughly evaluate sex-specific changes in response to the treatment. Despite these limitations, our study provides valuable insights into the mechanisms underlying DPP-4 inhibitors in chronic myocardial ischemia and serves as a foundation for further investigations in this field.

## Conclusion

SIT treatment produced a significant reduction in both interstitial and perivascular fibrosis in chronicaly ischemic myocardium. This remarkable decrease in fibrosis appears to be related to modulation of several pro-fibrotic signaling pathways in the ischemic myocardium, including JAK/STAT, SMAD, and mTOR. Moreover, SIT treatment resulted in a notable improvement in insulin signaling, as evidenced by decrease in the markers for insulin resistance and increase in insulin sensitivity signaling pathway in the ischemic myocardium. These combined effects of reduced fibrosis and enhanced insulin signaling could potentially account for the improved cardiac function observed in our prior studies and in human clinical trials. This promising outcome provides a clear direction for further investigations, both in human and animal studies, to delve deeper into the precise mechanisms of action and cardioprotective potential of DPP-4 inhibitors.

## Supporting information

S1 Checklist *PLOS ONE* humane endpoints checklist. (DOCX)

S1 Data (XLSX)

S2 Data (XLSX)

S3 Data (XLSX)

S4 Data (PPTX)
