# Supplementary material for: Dipeptidyl peptidase 4 inhibitor sitagliptin decreases myocardial fibrosis and modulates myocardial insulin signaling in a swine model of chronic myocardial ischemia
Source: PLoS One. 2024 Jul 29;19(7):e0307922. doi: 10.1371/journal.pone.0307922 (PMC11285952; doi:10.1371/journal.pone.0307922)
Supplement: S4 Data — (PPTX) [file pone.0307922.s005.pptx]

## Slide 1
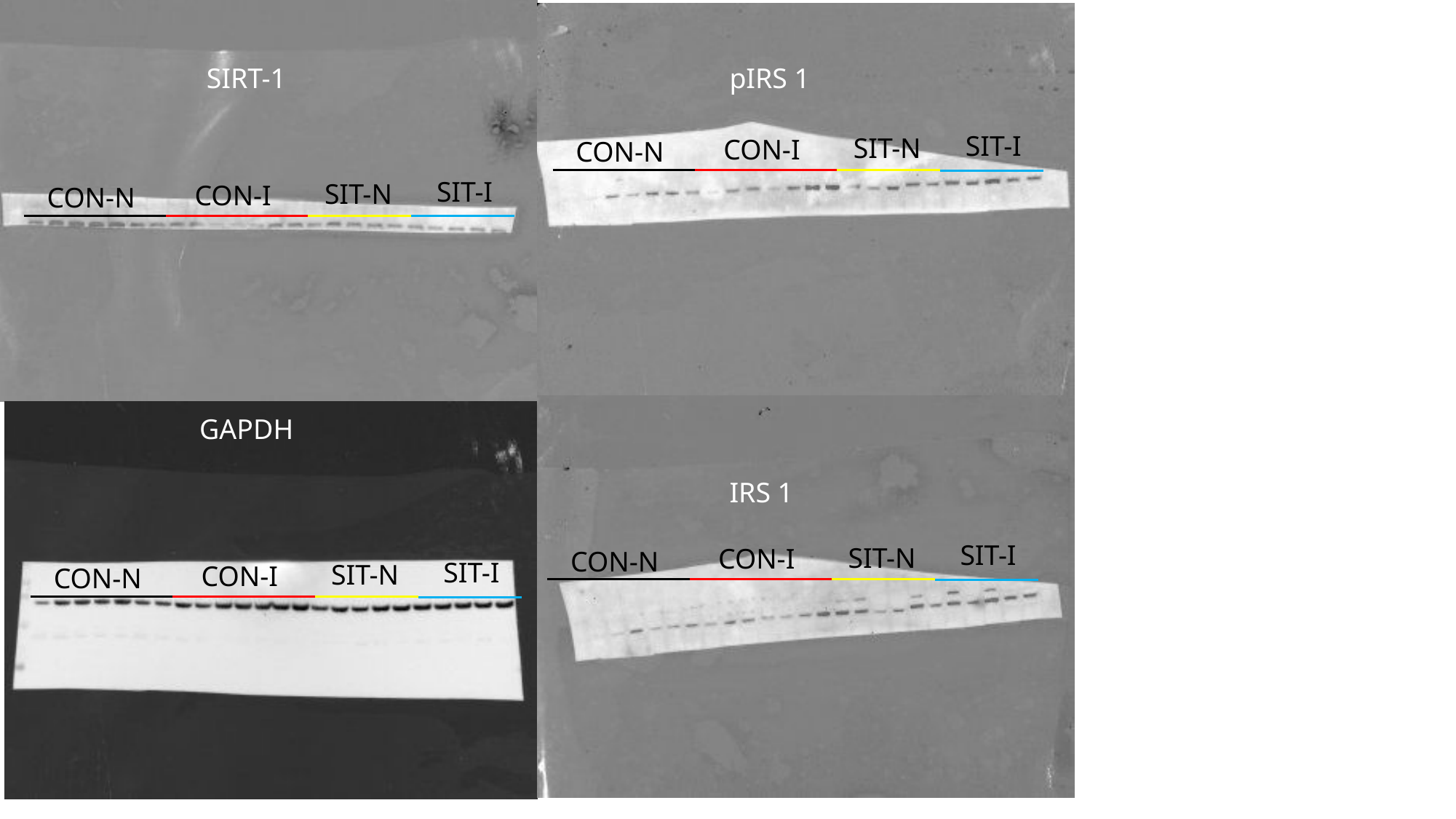

pIRS 1
SIRT-1
SIT-I
SIT-N
CON-I
CON-N
SIT-I
SIT-N
CON-I
CON-N
GAPDH
IRS 1
SIT-I
SIT-N
CON-I
CON-N
SIT-I
SIT-N
CON-I
CON-N

## Slide 2
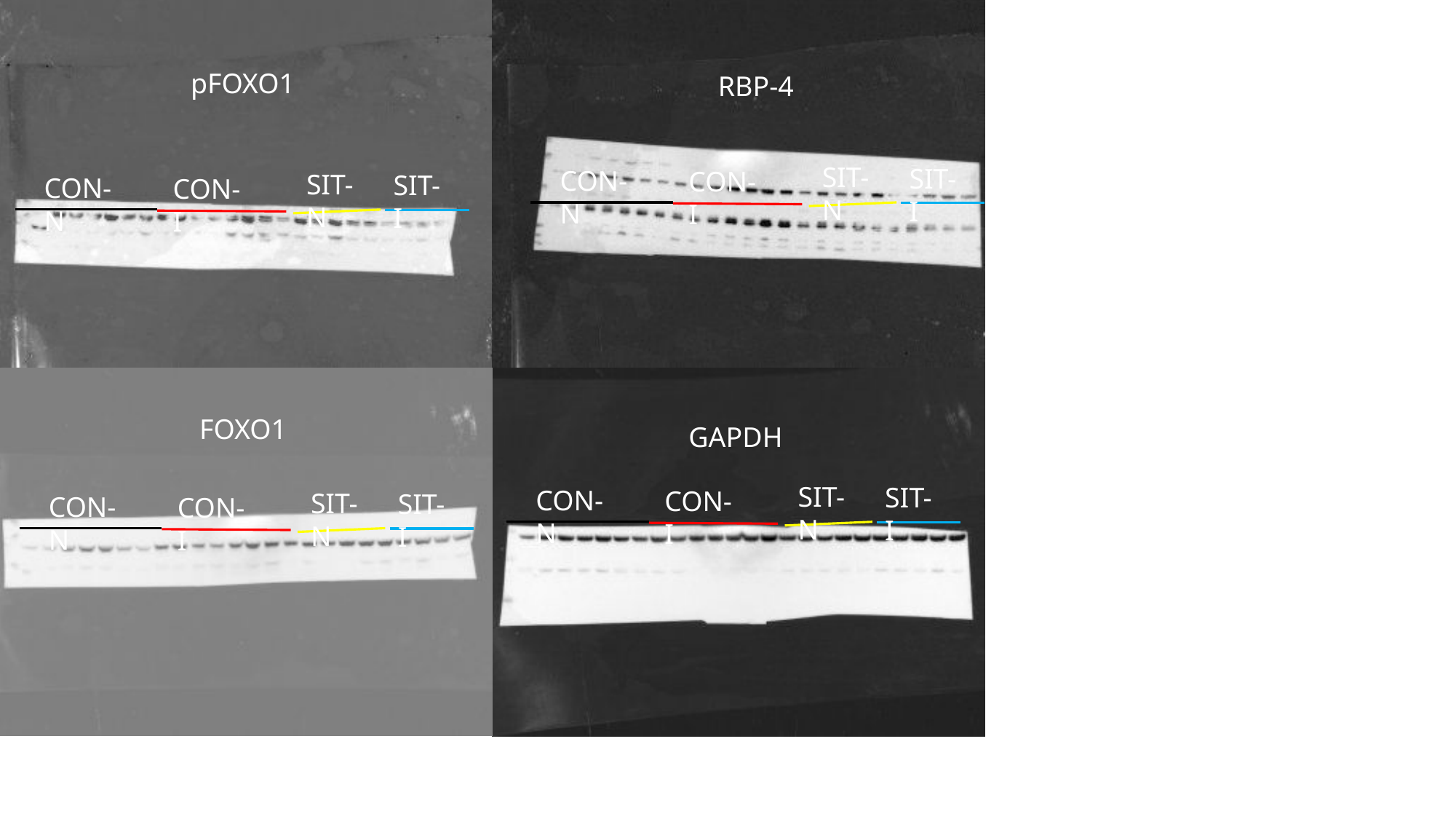

pFOXO1
RBP-4
SIT-N
SIT-I
CON-N
CON-I
SIT-N
SIT-I
CON-N
CON-I
FOXO1
GAPDH
SIT-N
SIT-I
CON-N
CON-I
SIT-N
SIT-I
CON-N
CON-I

## Slide 3
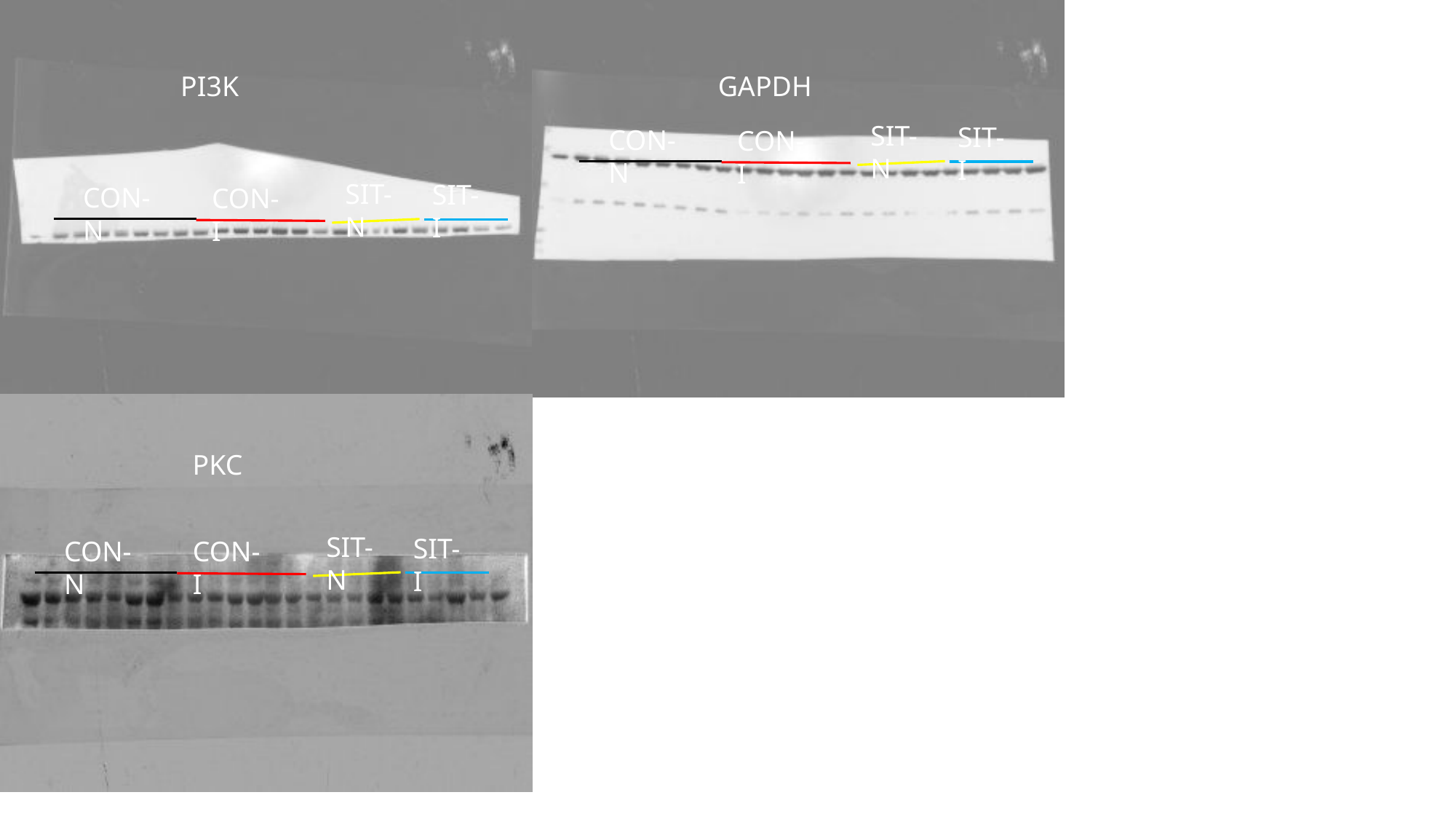

PI3K
GAPDH
SIT-N
SIT-I
CON-N
CON-I
SIT-N
SIT-I
CON-N
CON-I
PKC
SIT-N
SIT-I
CON-N
CON-I

## Slide 4
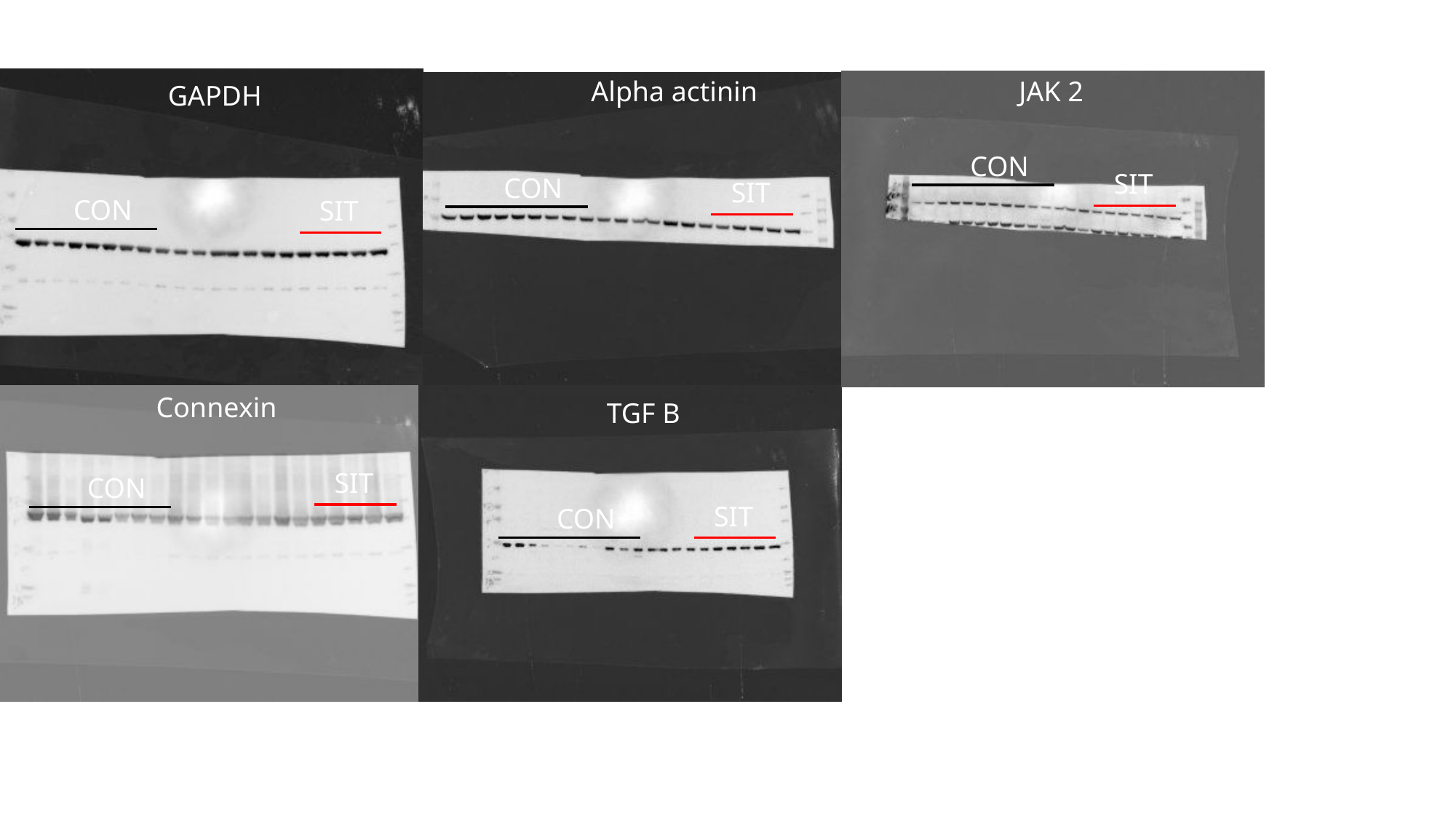

Alpha actinin
JAK 2
GAPDH
CON
SIT
CON
SIT
CON
SIT
Connexin
TGF B
SIT
CON
SIT
CON

## Slide 5
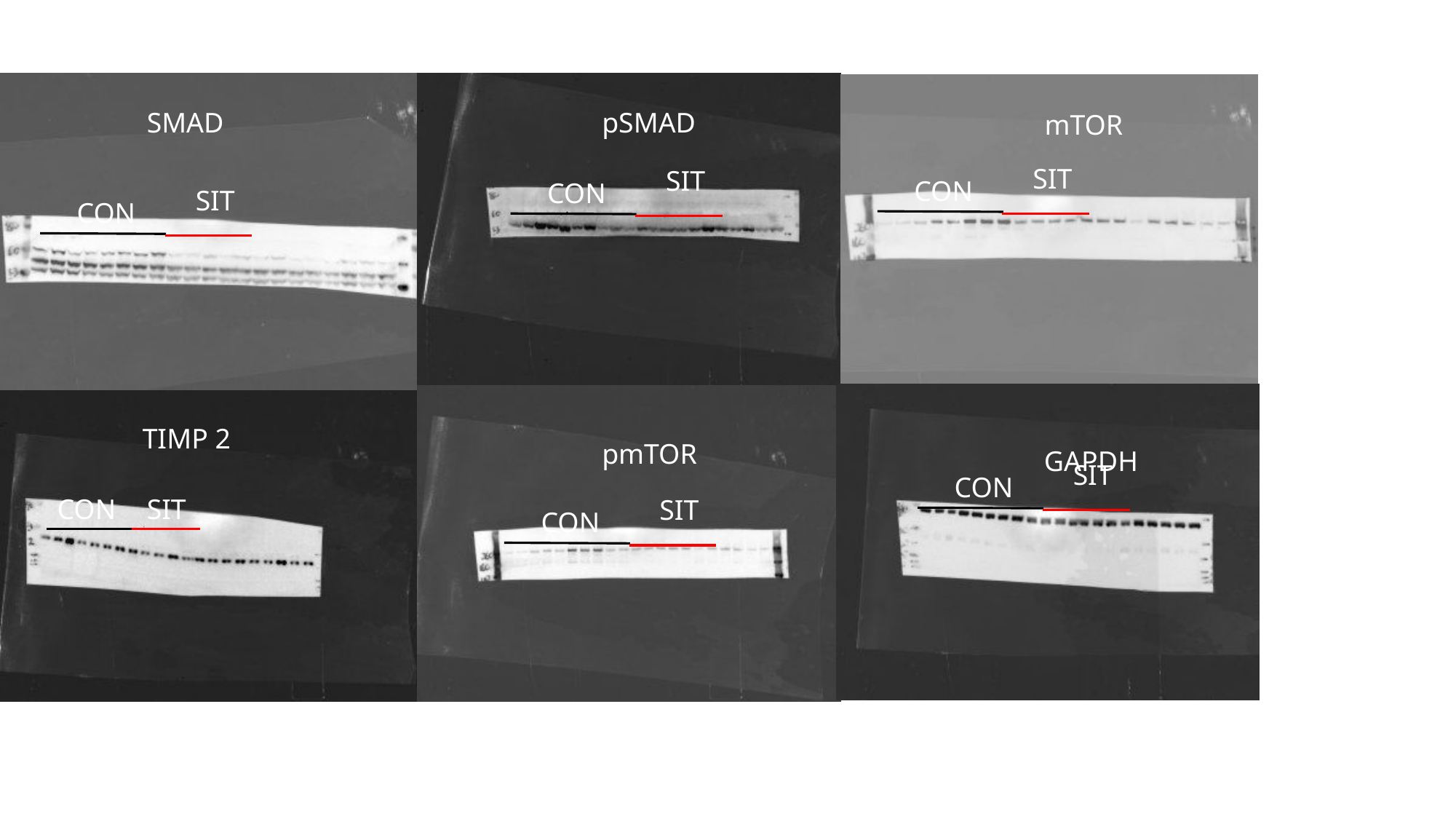

SMAD
pSMAD
mTOR
SIT
SIT
CON
CON
SIT
CON
Connexin
TIMP 2
pmTOR
GAPDH
SIT
CON
CON
SIT
SIT
CON

## Slide 6
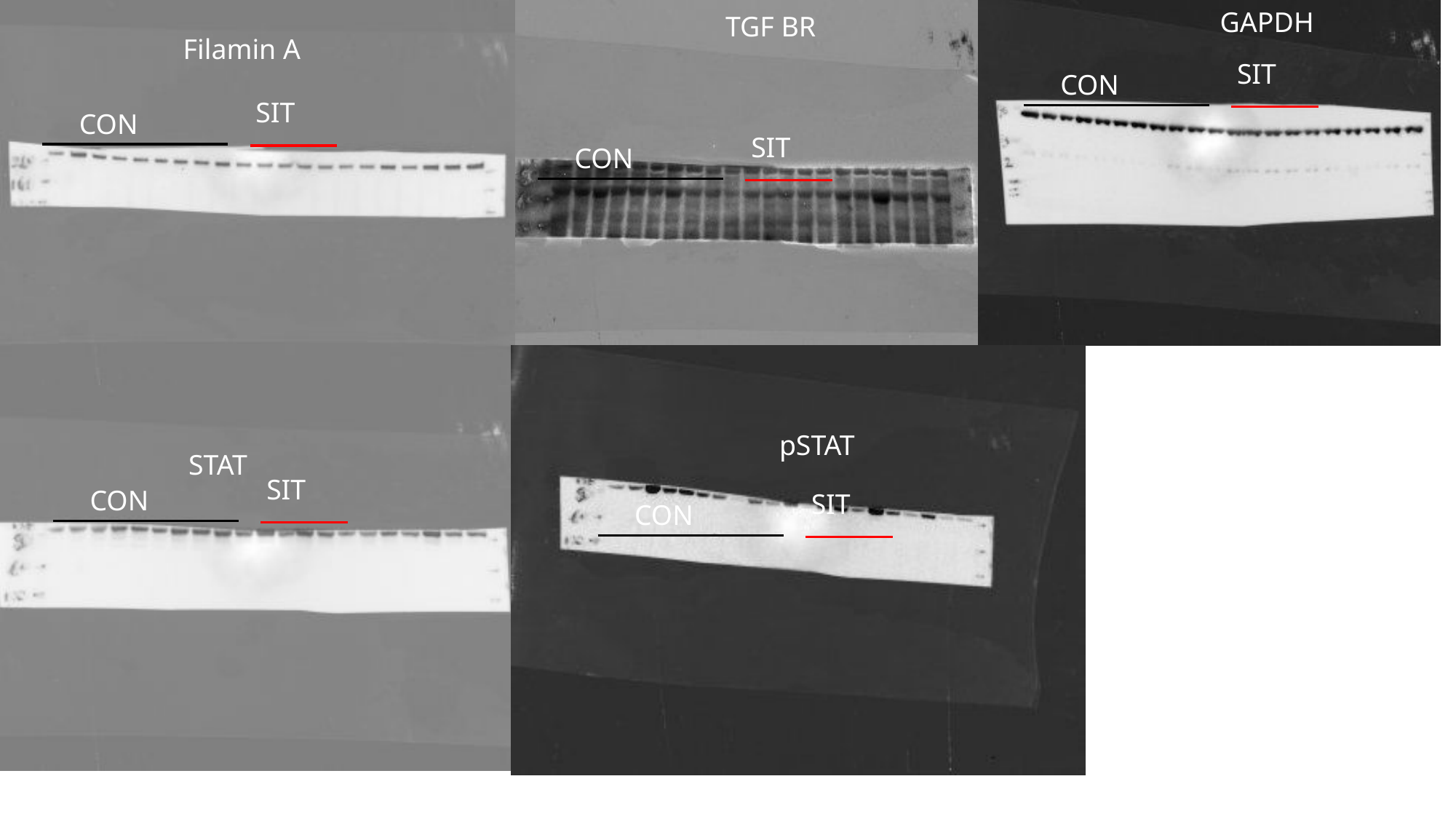

GAPDH
TGF BR
Filamin A
SIT
CON
SIT
CON
SIT
CON
pSTAT
STAT
SIT
CON
SIT
CON
